# Gold nanoparticles and their alternatives for radiation therapy enhancement

**DOI:** 10.3389/fchem.2014.00086

**Published:** 2014-10-14

**Authors:** Daniel R. Cooper, Devesh Bekah, Jay L. Nadeau

**Affiliations:** Department of Biomedical Engineering, McGill UniversityMontreal, QC, Canada

**Keywords:** nanoparticle, scintillator, radiation therapy, photodynamic therapy, photosensitizer, radiosensitizer

## Abstract

Radiation therapy is one of the most commonly used treatments for cancer. The dose of delivered ionizing radiation can be amplified by the presence of high-Z materials via an enhancement of the photoelectric effect; the most widely studied material is gold (atomic number 79). However, a large amount is needed to obtain a significant dose enhancement, presenting a challenge for delivery. In order to make this technique of broader applicability, the gold must be targeted, or alternative formulations developed that do not rely solely on the photoelectric effect. One possible approach is to excite scintillating nanoparticles with ionizing radiation, and then exploit energy transfer between these particles and attached dyes in a manner analogous to photodynamic therapy (PDT). Doped rare-earth halides and semiconductor quantum dots have been investigated for this purpose. However, although the spectrum of emitted light after radiation excitation is usually similar to that seen with light excitation, the yield is not. Measurement of scintillation yields is challenging, and in many cases has been done only for bulk materials, with little understanding of how the principles translate to the nanoscale. Another alternative is to use local heating using gold or iron, followed by application of ionizing radiation. Hyperthermia pre-sensitizes the tumors, leading to an improved response. Another approach is to use chemotherapeutic drugs that can radiosensitize tumors. Drugs may be attached to high-Z nanoparticles or encapsulated. This article discusses each of these techniques, giving an overview of the current state of nanoparticle-assisted radiation therapy and future directions.

## Introduction and background

Radiation therapy (XRT) is a critical component of the modern approach to curative and adjuvant treatment of cancers. XRT controls the growth of cancerous cells by bombardment with ionizing radiation, causing DNA damage by direct ionization or through generation of free radicals by ionization of water or oxygen molecules. Sufficient damage to DNA in this fashion can arrest cell growth and prevent metastasis. The primary drawback is collateral damage: there is little distinction in absorption between healthy and malignant tissues, and thus doses must be limited in order to mitigate unwanted damage to the tumor surroundings. External beam radiotherapy (EBRT) utilizes X-ray beams produced by orthovoltage units, or linear accelerators that may be spatially oriented, with beams shaped using multileaf collimators in order to maximize the specificity for the target. Distinct energy ranges are available for different EBRT targets: 40–100 kV (kilovoltage or “superficial” X-rays) for skin cancers or other exposed structures; as well as 100–300 kV (orthovoltage) and 4–25 MV (megavoltage or “deep” X-rays) for sub-surface tumors. Techniques such as 3-dimensional conformal and intensity-modulated radiation therapies have vastly improved the targeting capabilities of external beam therapy, but naturally there is still a strong desire to be able to further reduce the doses required for effective treatment. The SI derived unit for absorbed dose is the gray (Gy), equivalent to one joule of energy deposited by ionizing radiation per kilogram of matter (1 Gy = 1 J/kg = 1 m^2^/s^2^).

Brachytherapy, or internal radiotherapy, utilizes a radioactive source to provide a steady or pulsed dose of radiation to a small tissue volume. It is typically used for cervical, prostate, breast and skin cancers. Radioactive sources include ^125^I and^103^Pd, which produce γ rays of ~20–35 keV, ^192^Ir (γ rays, 300–610 keV), ^137^Cs (γ rays, 662 keV), ^60^Co (γ rays, 1.17 and 1.33 MeV), ^198^Au (γ rays, 410–1009 keV), ^226^Ra (γ rays, 190–2430 keV), and ^106^Ru which decays primarily through β^−^ emission at 3.54 MeV. Seeds of the listed materials can provide doses of up to 12 Gy/hour (high dose rate or HDR brachytherapy), though typical low dose rate (LDR) treatments amount to around 65 Gy over 5–6 days.

Heavy elements can be potent radiosensitizers (Kobayashi et al., 2010). It has been demonstrated that platinum-containing DNA-crosslinking drugs such as Cisplatin can enhance the effects of ionizing radiation through the “high Z effect,” or what has come to be known as Auger therapy. Heavy elements have significantly higher photoelectric cross-sections than soft tissue for sub-MeV energies, approximated for “X-ray energies” by the equation:

$$\sigma_{pe}\propto \frac{Z^{n}}{E^{3}}$$

where *σ_pe_* is the cross-section, *E* = *h*ν is the photon energy, *Z* is the atomic number, and *n* varies between 4 and 5 depending on the value of *E*. The photoelectric effect dominates below the electron rest energy of 511 keV, beyond which inelastic Compton scattering becomes more prevalent. As the photon energy decreases, it is no longer able to eject inner-shell electrons, producing the characteristic sawtooth pattern with K, L, and M edge structures. When ionized by X-ray or γ ray energy, mid- to high-Z elements (roughly Br and up) can produce a cascade of low-energy Auger electrons that can locally enhance the effective radiation dose (Kobayashi et al., 2010). Dense inorganic nanoparticles can also provide radiation dose enhancement that depends upon the composition and size of the particles, uptake of particles into cells, and the energy of the applied radiation.

## GNRT

Au nanoparticles have been under investigation for several years as possible agents for selective amplification of radiation dose in tumors, a concept called “gold nanoparticle-assisted radiation therapy” or GNRT (McMahon et al., 2008; Brun et al., 2009; Cho et al., 2009; Rahman et al., 2009; Van den Heuvel et al., 2010; Leung et al., 2011; Zhang et al., 2012). Reviews of this work can be found in Jelveh and Chithrani (2011), Butterworth et al. (2012), Jain et al. (2012), Babaei and Ganjalikhani (2014), Su et al. (2014).

The earliest studies used bulk or micro-sized gold to enhance radiation dose. Although this could be effective *in vitro* at a range of energies, micron-sized particles are not taken up well *in vivo*, even after intratumoral injection (Herold et al., 2000). Later experiments focused on Au nanoparticles or nanoclusters (1.9 nm diameter). When injected intravenously, these ultrasmall particles rapidly accumulated in cancer tissue, with 2.7 g Au/kg body weight resulting in 7 mg Au/g in tumor almost immediately after injection. Irradiation was performed about 60 s after injection, and with typical 250 kVp X-ray therapy, 1-year survival was 86% (compared to 20% with X-rays alone and 0% with gold alone) (Hainfeld et al., 2004). This result was followed by theoretical and experimental papers examining the mechanism of Au nanoparticle action as well as attempting to optimize Au particle concentration, size, and the energy and dose of applied X-rays (Cho et al., 2009; Zhang et al., 2009; Van den Heuvel et al., 2010; Leung et al., 2011).

### Improving GNRT by targeting

A significant problem with ultrasmall, nontargeted nanoparticles is rapid excretion by the kidneys. The amount of Au needed in early studies (>2 g Au/kg body weight) represents a very large amount of Au for human use. This is impractical, costly, and may cause toxicity. Achieving therapeutic levels in tumor with less delivered total Au is needed. In addition, irradiation in the mouse studies was performed immediately after particle injection. This is not practical in the clinic and may not work well in humans. Particles with longer circulation times, which can be delivered in multiple doses, are desirable for clinical applications. Optimizing the size, surface chemistry, and targeting of the Au nanoparticles may improve circulation times and accumulation in specific tumors.

**Figure 1:**
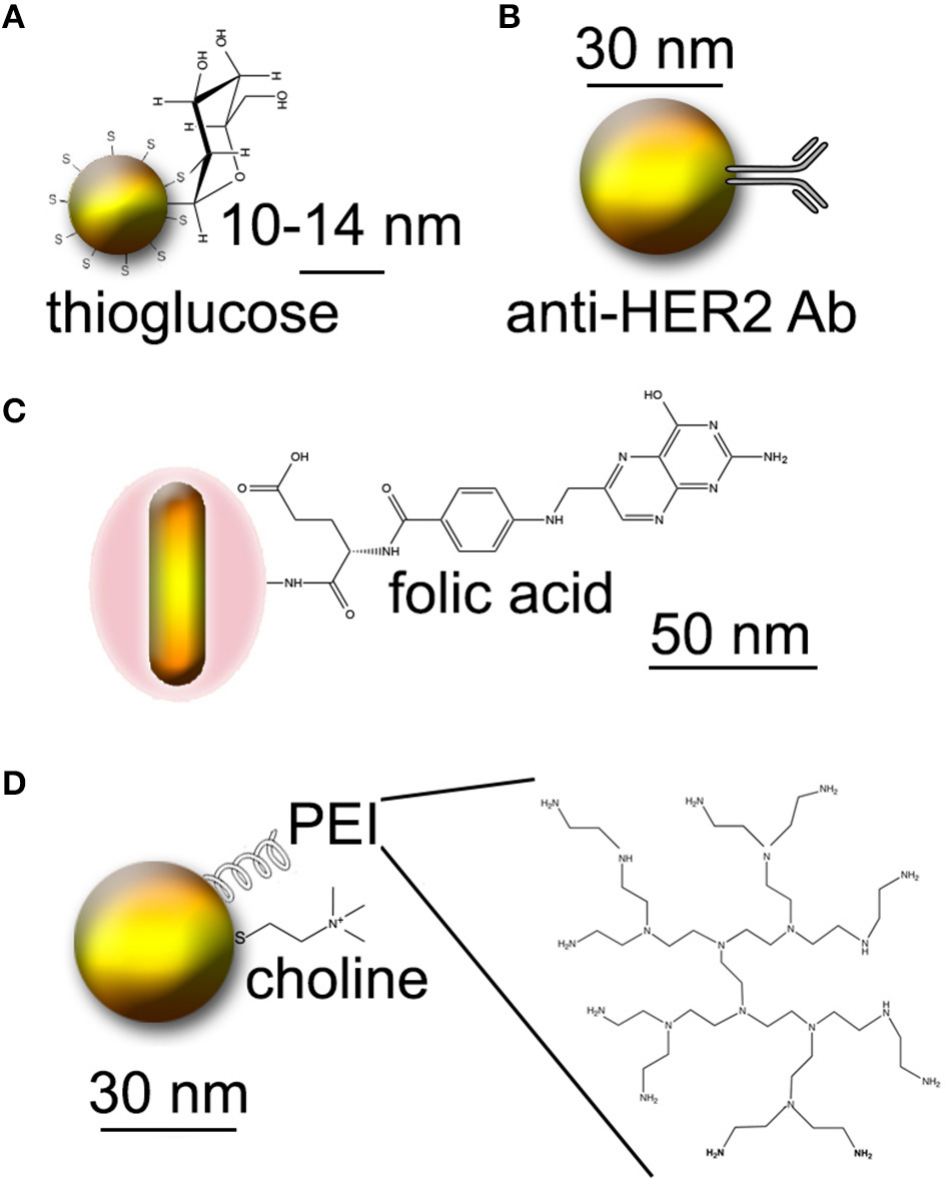
**Approaches to creating tumor-targeted Au nanoparticles**. Molecules not to scale. **(A)** Thioglucose-conjugated Au nanoparticles. **(B)** Au nanoparticles conjugated to Herceptin (anti-HER2 antibody). **(C)** Au nanorods conjugated to folic acid. **(D)** PEI-coated Au nanoparticles conjugated to choline.

The increased metabolic rate of tumors relative to normal tissue results in a high demand for glucose. Several studies have used thioglucose-conjugated Au nanoparticles (Figure 1A) in order to increase uptake by cancer cells. One study using ~14 nm Au demonstrated significantly increased uptake of thioglucose-conjugated particles by an ovarian cancer cell line after 8–96 h of incubation (Geng et al., 2011). A significant increase in inhibition was seen in the presence of 5 nM particles using 90 kVp or 6 MV X-rays; dose enhancement was significant relative to control beginning at 5 Gy and extending to 20 Gy, where all cells were inhibited even in the absence of particles. Another study compared cysteamine and thioglucose-coated 15 nM Au nanoparticles in breast cancer and normal breast cell lines (Kong et al., 2008b). Cysteamine-coated particles were taken up 3- to 4-fold more efficiently than glucose-coated particles. However, when applied to cells at concentrations that led to similar intracellular Au concentrations, glucose-coated particles led to increased radiosensitization relative to cysteamine-capped particles. Interestingly, radiosensitization by Au was not seen in a nonmalignant breast cell line, although the cells grew at the same rate as the cancer cells and took up an equal number of particles. The ability of ^167^Cs and ^60^Co sources to inhibit the cancer cells was also demonstrated in this paper.

The use of larger Au particles (57 nm and 84 nm) coated with thioglucose has been studied in another report (Song et al., 2013). These particles were taken up in equal numbers by HeLa cells. Surprisingly, unconjugated particles showed a greater radiosensitizing effect than thioglucose-conjugated particles, which the authors attributed to possible absorbance of singlet oxygen by the thioglucose shell.

Another study used the humanized anti-HER2 antibody Trastuzumab (Herceptin), PEGylated and conjugated to 30 nm Au particles, for delivery to MDA-MB-361 breast cancer (Chattopadhyay et al., 2013) (Figure 1B). Both *in vitro* and *in vivo* studies were performed. *In vitro*, an effective dose enhancement factor of 1.6 was seen in the presence of 2.4 mg/mL particles using 100 kVp X-rays. Delivery to MDA-MB-361 xenografts was done intratumorally, with ~0.8 mg total Au used (4.8 mg/g tumor). 11 Gy of 100 kVp image guided X-ray irradiation was performed 24 h after injection. This subtoxic dose led to a 46% reduction in tumor size relative to irradiation alone, with no damage to normal tissue or systemic toxicity.

Folic acid is another nutrient for which the need is increased in cancer cells. Conjugation to folate has been used for a wide variety of targeting applications for cancer and inflammatory diseases; a review may be found here (Low et al., 2008). Intra-operative tumor imaging using folate targeting has recently moved to the clinic for ovarian cancer (van Dam et al., 2011). In terms of GNRT, one study reported the use of silica-modified Au nanorods (~50 nm long) conjugated to folate (Huang et al., 2011) (Figure 1C). The rods were taken up by MGC803 human gastric carcinoma cells. 6 Gy of X-irradiation led to a 60% decrease in cell viability in the presence of 12.5 μM rods relative to cells without Au. The study also demonstrated uptake of the rods by MGC803 xenografts in nude mice, with contrast sufficient for X-ray imaging. No radiation experiments on animals were reported.

Cancers are also often distinguished by a lower pH than healthy cells due to hypoxia and resulting anaerobic metabolism within tumors. The pH-sensitive pHLIP peptide was used in one study to target Au nanoparticles to mice bearing HeLa tumors (Yao et al., 2013). Although radiation was not performed, accumulation of Au in tumors sufficient for radiotherapy enhancement was demonstrated, with the stated goal of using the construct for this purpose.

Prostate cancer is an excellent target for nanoparticle-enhanced radiation, since it is often treated by brachytherapy and is accessible to intratumoral injection. Choline is a ubiquitous molecule in all cells for which overactivity of processing enzymes (choline kinase) has been found in prostate tumors. One study reported development of polyethylene imine (PEI)- and choline-conjugated Au nanoparticles for the purpose of targeting prostate cancer for GNRT (Razzak et al., 2013) (Figure 1D). While no radiation experiments were performed in this study, favorable pharmacokinetics were shown in mice.

These studies illustrate that targeted GNRT remains an area requiring substantial further investigation. While one or more targeting agents may be conjugated to Au nanoparticles, and while these may improve delivery *in vitro* and even *in vivo*, it is not fully established whether these formulations improve tumor response to radiation therapy. The possibility that an organic shell can absorb reactive oxygen species deserves further inquiry. The size of the nanoparticles used, the density of targeting ligands, and the delivery method (IV, intratumoral, concentration, timing) all need to be optimized. The good news is that many of these formulations use FDA-approved ingredients, some of which are currently in the clinic for imaging. Optimization in animal studies should thus lead to rapid clinical translation.

### Improving GNRT by addition of photothermal therapy

Hyperthermia therapy is a minimally invasive treatment in which the temperature is increased locally (up to 44°C) to kill malignant cells. Even though hyperthermia can kill cells on its own, it is more often used in combination with other treatments such as radiotherapy or chemotherapy (Wust et al., 2002); such combinations are in clinical trials (Vernon et al., 1996; van der Zee et al., 2000; Zagar et al., 2010). An increase in nuclear damage is one of the mechanisms through which cells are radiosensitized after hyperthermia (Wust et al., 2002; Kampinga, 2006). In addition, the higher temperature causes dilation of the blood vessels, increasing oxygenation of the tumor (Griffin et al., 1996; Song et al., 2009). Since oxygen is a potent radiosensitizer, it can increase the damage to the tumor through generation of free radicals.

Methods to locally heat the tumor region include high intensity focused ultrasound (HIFU), microwave heating, magnetic hyperthermia, and photothermal therapy. In photothermal therapy, a light source (usually infrared) is used to deliver heat to the tumor. Such approaches are difficult to target, but delivery of nanomedicines to the tumor could improve the local heating profile. Most studies have looked at gold nanoparticles and nanorods for this purpose, because exposure of Au nanoparticles to IR light causes a local temperature increase due to surface plasmon resonance (El-Sayed et al., 2006; Huang et al., 2006; Gobin et al., 2007; Hainfeld et al., 2010; Verma et al., 2014). By modifying the size and shape of these nanoparticles, the resonance peak can be tuned to different wavelengths in the IR.

Delivery of gold followed by heating and ionizing radiation has proved to be a promising approach in pre-clinical studies. Gold nanoshells with a 120 nm silica core and a 12–15 nm shell were used in one study (Diagaradjane et al., 2008) to treat a murine xenograft model of human colorectal cancer. Localized hyperthermic treatment followed 5 min later by a 10 Gy X-ray dose were given 20–24 h after IV delivery of the nanoshells. The tumor volume doubling time was significantly greater for the treated mice. Two mechanisms were identified as contributing to the treatment's efficacy: an increase in perfusion resulting in a decrease in tumor hypoxia, and vascular collapse in the tumor due to accumulation of nanoparticles around the blood vessels. Another study confirmed these results using similar gold nanoshells in two murine breast cancer models (Atkinson et al., 2010).

Another study used gold nanorods modified with silica and conjugated to folic acid (Huang et al., 2011) to test the effects of photothermal and radiation therapy on MGC803 gastric cancer cells. The two treatments were tested separately and not combined. For the radiation treatment, a 6 MeV source was used to deliver doses of up to 10 Gy. Cell survival with radiation decreased in a concentration-dependent fashion with nanoparticle addition; the particles were non-toxic in the absence of radiation. Photothermal therapy consisted of 3 min of irradiation with a 30 mW, 808 nm laser source. Apoptosis was seen after treatment in the presence of the gold particles.

A recent study (Hainfeld et al., 2010) calculated the radiation dose required to control 50% of tumors (TCD50) in a mouse squamous cell carcinoma model. Gold nanoparticles were delivered intratumorally, and 24 h later the tumor was heated to 48°C for 5 min at 1.5 W/cm^2^ followed by X-ray irradiation at 100 kVp (7.5 Gy/min). TCD50 was reduced from 55 Gy to less than 15 Gy.

These studies illustrate one of the biggest problems of the approach, which is the need for simultaneous delivery of heating and radiation, which poses logistic problems in the clinic (Wust et al., 2002). Other drawbacks include a lack of specificity and the difficulty of heating deep tumors.

## Alternatives to gold: bismuth and iron

Alternatives to Au are being sought that are more effective and/or less costly. Bismuth (Bi, *Z* = 83) and platinum (Pt, *Z* = 78) have been shown in at least one theoretical study to yield a dose enhancement factor higher than Au, with Bi being the highest. Dose enhancement is predicted to increase with decreasing nanoparticle size, because the smaller nanoparticles accumulate closer to the nucleus, where they can cause the greatest damage. The dose enhancement is also expected to be greater when the average energy is close to the K-edge of the element (Ngwa et al., 2010; Hossain and Su, 2012). A radiochromic dosimeter was used in another study to experimentally measure the dose enhancement of bismuth oxide (Bi_2_O_3_) nanoparticles. Using a 100 kV X-ray source and an irradiation dose of 10 Gy, the radiation dose in a water-equivalent matrix doped with 0.5 mM of 50 nm Bi_2_O_3_ nanoparticles was >80% higher than in the control compartment (Alqathami et al., 2013). Another study (Zhang et al., 2014) looked at the dark toxicity, biodistribution, and radiation effects of bismuth selenide (Bi_2_Se_3_) nanoplatelets in cell lines and mice. The platelets were not significantly toxic to either cells or mice, with over 93% of the Bi cleared from the body 90 days after treatment. Significant radiation dose enhancement was observed after irradiation doses of up to 8 Gy.

Gadolinium (Gd, *Z* = 64) represents another alternative to gold nanoparticles. In addition to having a relatively high atomic number, Gd is already routinely used as a contrast agent in MRI. Gd_2_O_3_ core nanoparticles encapsulated in a polysiloxane shell have shown potential as an image guided radiotherapeutic tool in a gliosarcoma rat model (Le Duc et al., 2011). Accumulation of the nanoparticles in the tumor after saphenous vein injection was demonstrated using MRI, and the tumor-bearing rats were treated with microbeam radiation therapy, with a significant increase in survival in the nanoparticle-treated group. Another study using a rat brain tumor model confirmed that ultra-small Gd-based nanoparticles accumulate in brain tumors after IV injection (Miladi et al., 2013).

Magnetic particles such as iron oxide may also be used for combined hyperthermia and radiation. By using an alternating magnetic field to excite magnetic nanoparticles, local temperature increases can be achieved. The advantages of iron oxide include low toxicity, ease of synthesis, and the ability to perform image guidance using MRI. Dextran-coated iron oxide has been shown to reduce tumor growth in a syngeneic mouse breast cancer model when hyperthermia and radiation were combined (Giustini et al., 2011).

Several studies have looked at radiosensitization properties of iron oxide nanoparticles. Using 6 MeV X-rays on a human prostate carcinoma cell line (DU145), 1 mg/ml of Fe_3_O_4_ nanoparticles resulted in a dose enhancement factor of approximately 1.2 (Khoei et al., 2014). Another study suggested that superparamagnetic iron oxide nanoparticles (SPIONs) can radiosensitize tumor cells by catalyzing ROS formation. Uncoated, citrate-coated, or malate-coated SPIONs were added to MCF-7, 3T3, and Caco-2 cells. Uncoated SPIONS caused dark toxicity, with no increase in ROS upon 1 or 3 Gy irradiation. In contrast, coated SPIONS were non-toxic in the absence of radiation, but resulted an increase of up to 300% in the fluorescence intensity of the ROS reporter dichlorofluorescein diacetate (DCF-DA) (Klein et al., 2014).

## Scintillating nanoparticles for radiation/photodynamic “hybrid” therapy

### Introduction and concept

**Figure 2:**
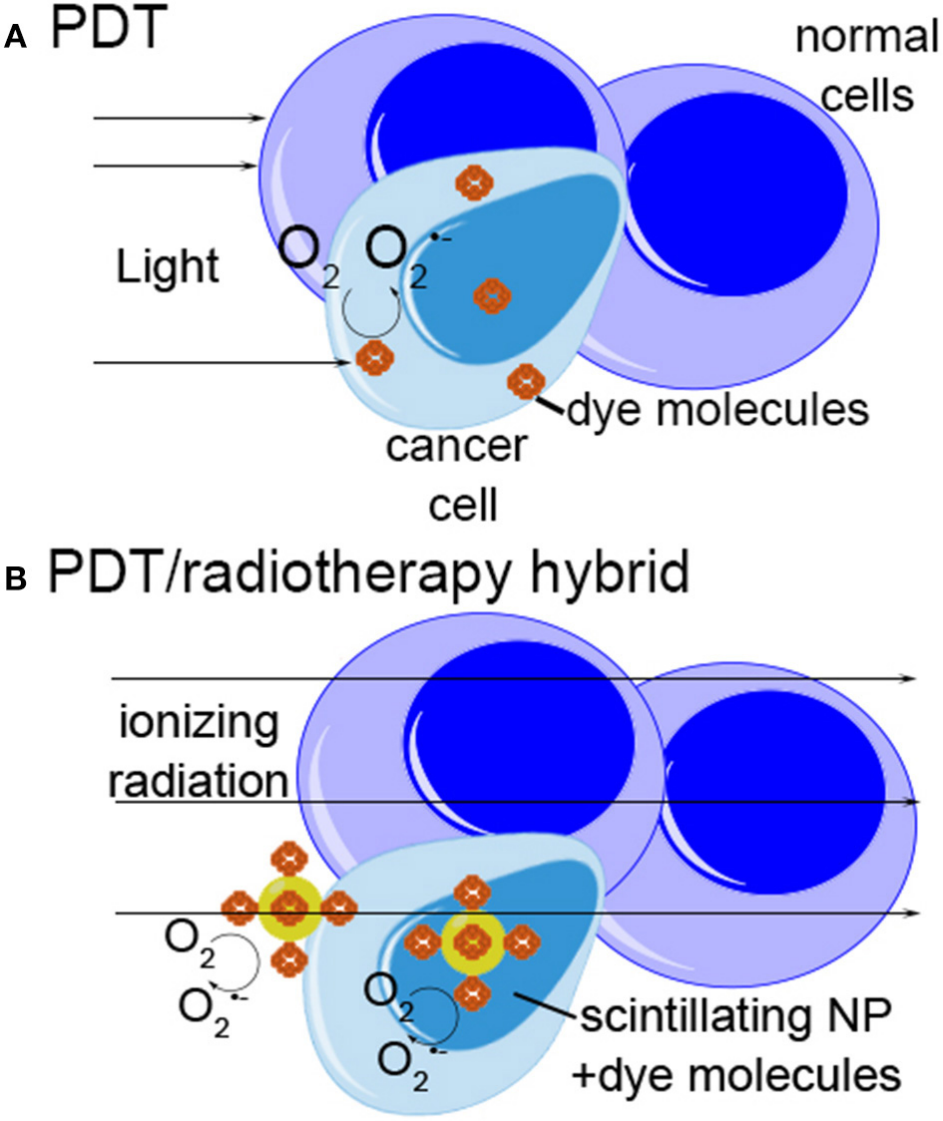
**Schematic of photodynamic therapy and radiotherapy-photodynamic therapy “hybrid” approach. (A)** In photodynamic therapy, photosensitizer dye molecules collect preferentially in malignant or inflamed tissue. Light is used to excite the dye, generating reactive oxygen species which lead to cell killing. **(B)** In the “hybrid” approach, ionizing radiation is used to excite scintillating nanoparticles, which may be located deep within tissue. The nanoparticles transfer energy to attached photosensitizer molecules, generating ROS and killing cells by the same mechanism as photodynamic therapy.

**Figure 3:**
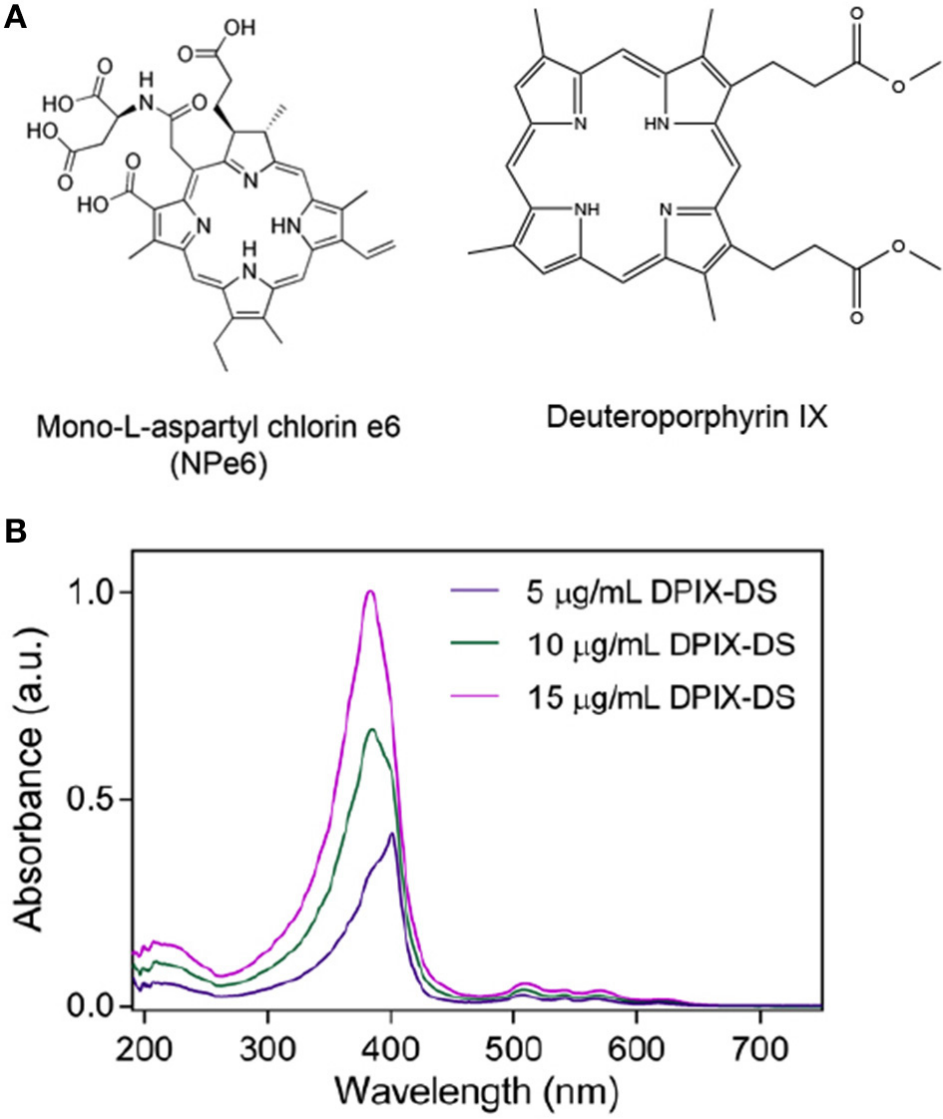
**Photosensitizers. (A)** Typical photosensitizer structures: mono-L-aspartyl chlorin e6 (Talaporfin sodium), a PDT drug that can be isolated from algae or green plants (approved in Japan and in Phase III trials in the U.S.); deuteroporphyrin IX, a candidate photosensitizer with several possible derivatives. **(B)** Absorbance spectra of different concentrations of deuterophorphyrin IX disulfonic acid (DPIX-DS). Note the strength of the Soret band (UV-blue) compared to the peaks in the redder regions.

A 2006 study proposed a new approach to nanoparticle-based therapies aiming to combine and enhance the effects of radiation therapy and photodynamic therapy (PDT) through the use of scintillating nanoparticles conjugated to photosensitizer molecules (Chen and Zhang, 2006). The concept is simple: attach a dye used for PDT to a nanoparticle that emits light when excited by therapeutic radiation (scintillates). If the scintillation emission overlaps the absorbance spectrum of the dye, the dye will generate singlet oxygen as it does with light-excited PDT (Figure 2). Many conventional photosensitizers are based on naturally occurring porphyrin, chlorin, and bacteriochlorin structures comprised of highly conjugated heterocyclic macrocycles (Figure 3A). These molecules have a strong absorbance peak in the UV to blue range (Soret band) as well as numerous weaker peaks in the visible (Figure 3B).

This idea has attracted significant attention over the past few years (Cheng and Lo, 2011) because it promises to combine the tissue penetration depth of radiation with the efficacy and benign side effect profile of PDT. PDT results in less damage to normal tissue than does radiation therapy; does not induce scarring; may be repeated multiple times; and may spark immune responses that help destroy the tumor. However, because of the limited tissue penetration depth of visible and even near-IR light, this therapy is restricted to only the most superficial cancers, such as non-melanoma skin cancer and bladder cancer.

The challenge is to develop stable, nontoxic nanoscintillators that may be delivered to cells. Several varieties of doped insulator and semiconductor nanoparticles have been proposed to fill this role. While scintillation has been demonstrated for CdSe/ZnS quantum dots (Létant and Wang, 2006), they have poor radiation hardness and degrade rapidly under γ ray exposure (Withers et al., 2008). As the toxicity of these materials is also primarily related to their chemical degradation, alternatives are necessary. The development of such alternatives is mostly in early stages. Although many of the approaches to surface chemistry and targeting that have been used for gold could be applied to these other materials, this has not yet been attempted. Some of the materials also show specific chemical challenges as we will discuss in Section Biocompatibility of lanthanide-based materials.

### Scintillation

Scintillation, or radioluminescence (RL), is the process whereby a material, referred to as a scintillator, produces light upon interaction with ionizing radiation. Inorganic nanoparticles (NPs) doped with lanthanides present an attractive, radiostable alternative to quantum dots for scintillation.

#### Introduction to lanthanide luminescence

Lanthanides are well known for the luminescence of their trivalent cations, which emit primarily through phosphorescence resulting from electronic transitions within the *4f* shell (Bünzli and Eliseeva, 2010). Because these transitions are “forbidden” by Laporte's parity selection rule (formally prohibiting electric dipole transitions between states that conserve parity), they have low absorption cross-sections and their photoluminescence is commonly sensitized by Ce^3+^ (for downconversion, with Tb^3+^ acceptor) or Yb^3+^ (for upconversion, with Tm^3+^, Er^3+^, and Ho^3+^ acceptors), though more complex combinations of lanthanides are certainly possible. The efficiency of both processes benefits from a low phonon energy host, though is of increasing importance for lower energy transitions. In the case of upconverting NPs, hexagonal phase (β phase) NaYF_4_ or isostructural NaGdF_4_ are generally the preferred host materials.

The mechanism of cerium luminescence is distinct from most other lanthanides. Neutral cerium has a [Xe]4f^1^5d^1^6s^2^ electronic configuration; in solution or in solid hosts, the +3 or +4 oxidation states are the most common. Only the +3 state is luminescent, though the +4 state also has important implications for redox activity. In the +3 state, the 6*s* and 5*d* electrons are lost, leaving one optically active electron in the shielded 4*f* shell. Fluorescence (Δ*S* = 0) arises from parity-allowed, high oscillator strength 4*f*-5*d* transitions. Because the 5*d* orbitals are external, these transitions are sensitive to the crystal field, and vary in energy across a substantial range depending on the host material (Dorenbos, 2000).

Cerium-doped lanthanum fluoride (Ce_*x*_La_1−*x*_F_3_) shows luminescence in the UV-blue (corresponding well to the Soret band) and so is a likely candidate for useful energy transfer to photosensitizers.

#### Mechanisms of scintillation

RL mechanisms of bulk Ce_*x*_La_1−*x*_F_3_ crystals were elucidated in the late 80 s and early-to-mid 90 s as candidates for radiation detection purposes (Moses and Derenzo, 1989, 1990; Wojtowicz et al., 1992, 1994; Lempicki et al., 1993; Moses et al., 1994; Rodnyi et al., 1995). Though the scintillation was found to be significantly faster than commonly used scintillators at the time (BGO, CsI:Tl, NaI:Tl) on a per-photon basis, the overall light output was found to be unexpectedly weak, with variable luminescence that was significantly dependent on the quality of the crystal and the presence of defects. This variability precluded their use as reliable detectors for the most part, at least compared to other options being developed concurrently, such as PbWO_4_.

The general process of activator-based scintillation occurs in three steps: first, conversion of absorbed ionizing radiation energy into electronic-lattice excitations (electron-hole pairs and/or excitons), followed by transfer of the excitation energy to the emitting centers and then luminescence. The overall scintillation efficiency is given by the product of the individual efficiencies:

η=βSQ,  0≤η,β,S,Q≤1

where β, the efficiency of the conversion process, encompasses the fraction of absorbed energy lost to optical phonons, *S* is the efficiency of the transfer process, and *Q* is the luminescence quantum yield of the emitting center. The overall light output *L* (in photons/MeV) is given by:

$$L=n_{e-h}\eta =\frac{10^{6}}{2.3E_{g}}\beta SQ$$

where *n_e−h_* is the number of *e-h* pairs or excitons that are generated per MeV of absorbed radiation, discounting losses to optical phonons, and *E_g_*is the band gap of the host (in eV). The factor of 2.3 is related to the derived minimum incident photon energy required to generate a single *e-h* pair (Robbins, 1980), ξ_min_ = 2.3 *E_g_*, and so *n_e−h_* = *E*/2.3 *E_g_* where *E* is the energy of the incident photon, in this case 1 MeV = 10^6^ eV.

Low phonon energy hosts such as LaF_3_ tend toward higher values of β, while the transfer process *S* is relatively inefficient compared to pentaphosphate or orthophosphate hosts (Lempicki et al., 1993). The β and *S* mechanisms of Ce_*x*_La_1−*x*_F_3_ were determined to consist of three distinct processes that have different relative contributions depending on the value of *x*: (i) direct excitation of Ce^3+^ by X-rays or secondary electrons, (ii) ionization of Ce^3+^ followed by electron capture and formation of bound excitons, or (iii) energy transfer to Ce^3+^ from lattice excitations of the bulk matrix. At lower concentrations of Ce^3+^, up to *x* ~ 0.5, mechanism (iii) dominates the scintillation response. At higher doping levels, mechanism (i) is predominant, accounting for a large fraction of the light output in CeF_3_. It has recently been demonstrated that co-doping single crystals of YPO_4_:Ce^3+^ with Pr^3+^, which act as electron traps, can improve scintillation efficiency by minimizing the influence of defects as well as mitigating the effects of damage caused by prolonged irradiation (Moretti et al., 2014).

#### Nanoscintillators

A number of reports have investigated the scintillation response of Ce_*x*_La_1−*x*_F_3_ nanocomposites, where small NPs (~10 nm in diameter) are cast into oleic acid or polymer matrices with consistencies ranging from liquid to waxy. In initial studies, nanocomposites exhibited photopeaks for ^137^Cs, ^241^Am, and ^57^Co irradiation (McKigney et al., 2007a,b). Most recently, a modest scintillation response (compared to a BC-400 polyvinyltoluene detector) has been shown for 25% NP-loaded composites exposed to several sources: ^22^Na (3.22 μCi), ^60^Co (3.78 μCi), ^137^Cs (31.9 μCi), ^241^Am (9.09 μCi), and ^252^Cf (5.03 μCi) (Guss et al., 2013). For radiation detection purposes, fast lifetimes are typically preferred, whereas for bioconjugates, short lifetimes may preclude efficient energy transfer if it is outcompeted by luminescence or quenching processes.

While the scintillation of cerium in simple fluoride or phosphate hosts is well studied, it is just one of a number of possible scintillation mechanisms. In the late 2000 s, a number of reports were released discussing the possibilities and limitations for nanoscintillators in a broad sense, including the demonstration of a few crucial nanoscale phenomena (Klassen et al., 2008, 2009; Dujardin et al., 2010; Kortov, 2010). Several research groups are now engaged in the development of a wider variety of nanoscintillators, either through adaptation of known scintillating materials to the nanoscale, or through the creation of novel compositions. Many of these are based on luminescent “activator” dopants, including lanthanides (Ce^3+^, Pr^3+^, Tb^3+^, or Eu^2/3+^). RL spectra have been published for a number of fluoride nanoscintillators, including powdered LaF_3_:Eu (~4.4 nm), BaF_2_:Ce (~10 nm), and CaF_2_:Eu (~18 nm) NPs under excitation by a 40 kV Bullet X-ray tube and CaF_2_:Eu^3+^ excited by a 1 μCi ^241^Am source (*E*_α_ = 5.5 MeV, *E*_γ_ = 60 keV) (Jacobsohn et al., 2011). The authors suggest that in such doped ionic crystals, where the diffusion length of *e-h* pairs may be up to 100 nm, it is conceivable that scintillation yields may be limited by the physical dimensions of the NPs or by the total number of activators. The same group has also compared the effects of undoped LaF_3_ shell thickness on the photoluminescence *vs*. RL of LaF_3_:Eu NPs (Jacobsohn et al., 2010). The undoped shells act as a passivating barrier that is transparent to both optical excitation and emission, and PL efficiency was found to increase in a roughly linear fashion as a function of overall NP size as additional shells were added. With X-ray excitation, the shells were found to increase RL efficiency up to a shell volume of roughly twice the core volume, beyond which the light yield decreased with additional shell thickness. This was attributed to the increased undoped volume decreasing the probability of radiative recombination within the Eu-doped core volume, and suggesting that the diffusion length of carriers in LaF_3_ to be relatively short.

Indeed, the luminescence of core-only activator-based nanoscintillators has been found to be size-dependent in some cases. One study demonstrated a considerable broadening of Eu^3+^ emission lines in progressively smaller Gd_2_O_3_ NP hosts as compared to bulk crystals, attributed to increasing crystal field fluctuations in the smaller NPs (Dujardin et al., 2010). A number of physical mechanisms potentially influencing nanoscintillators are described in the report, including structural effects, surface effects, quantum confinement, and dielectric confinement. Also shown was a significant difference in the RL spectra of bulk *vs*. nanoscale CeF_3_ samples. Intriguing scintillation behavior from LuBO_3_:Ce nanocrystals has been reported, with a considerable dependence on the NC dimensions (Klassen et al., 2008, 2009). NC grain sizes were controlled by altering annealing temperatures, and scintillation yields were found to increase dramatically for NCs ~95 nm in diameter, with roughly three times the intensity of NCs either 25 nm larger or smaller. This is in contrast to LuF_3_:Ce NPs in the same size range, which exhibited a monotonic size dependence.

**Figure 4:**
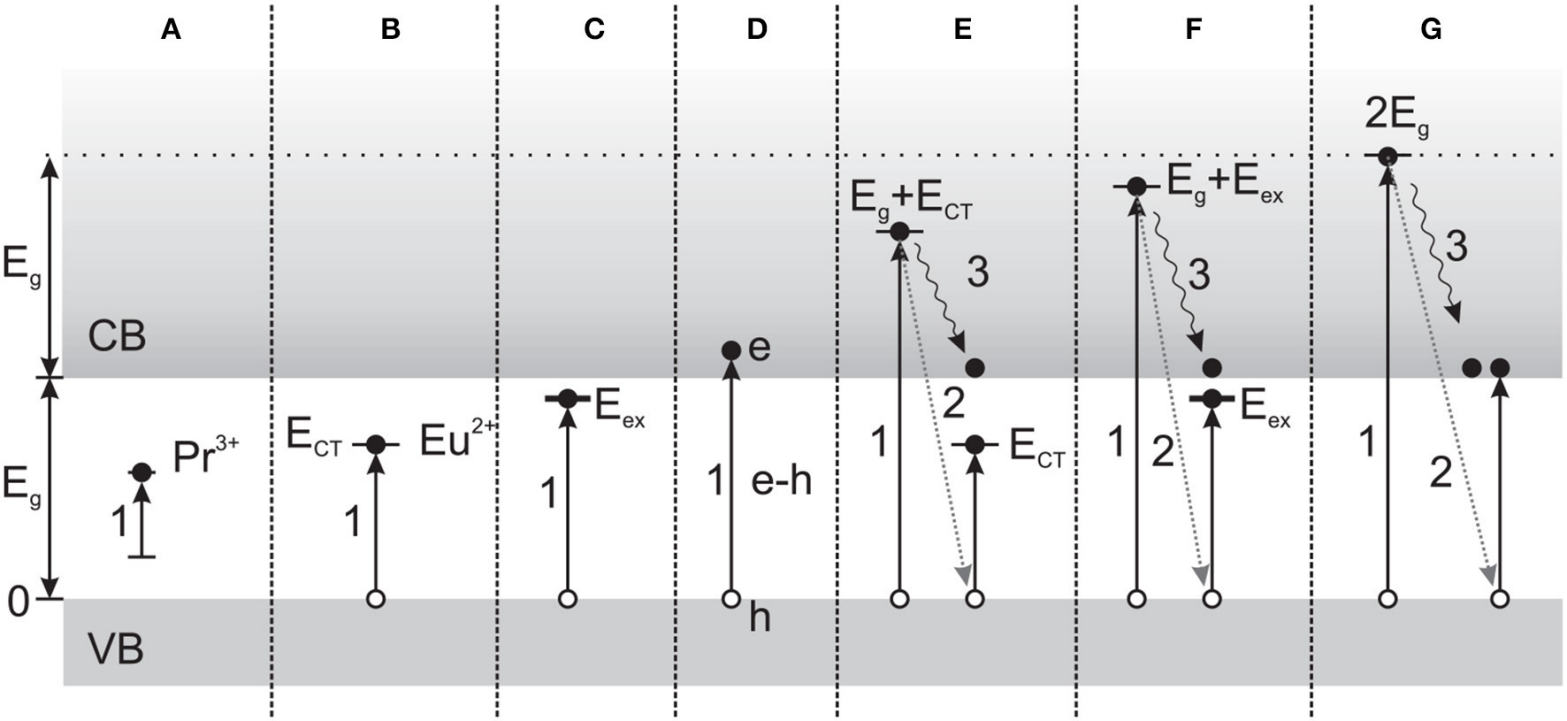
**Mechanisms of scintillation in Pr^3+^ or Eu^3+^-doped LaPO_4_, depending on excitation energy. (A)** Intracenter (direct) excitation of Ln activators. **(B)** Excitation by charge transfer from O^2-^ to Eu^3+^. **(C)** Direct exciton formation. **(D)** Creation of e-h pairs. **(E)** Excitation multiplication, with secondary excitation as in **(B)**. **(F)** Excitation multiplication, with secondary excitation as in **(C)**. **(G)** Photons with *E* > *E_g_* can result in excitation multiplication involving the creation of secondary e-h pairs. Arrows: (1) Transition due to photon absorption. (2) Energy exchange due to inelastic scattering on valence band electrons, and (3) relaxation of primary electrons. (Reprinted with permission from Malyy et al., 2013).

Synthesis techniques and post-synthesis processing affect the size, crystallinity, and dopant distribution of nanostructures. The role of post-synthesis annealing on NCs was recently investigated with LaPO_4_:Eu and LaPO_4_:Pr (Malyy et al., 2013), as well as LuPO_4_:Ce (Vistovskyy et al., 2014). In the case of LaPO_4_:Ln, annealing was used to increase the size of the NCs, also resulting in a change of lattice symmetry above ~500°C. The subsequent effects on excitation processes over the range of 4–40 eV are described in some detail. Across the energy range investigated, the distinct mechanisms include intracenter excitation, charge transfer excitation, exciton or e-h pair creation, electronic excitation multiplication (*E* > 2 *E_g_*), or combinations (Figure 4), and different sensitivities were shown for the two activators—the first stage of Eu^3+^ recombination involving electron capture, in contrast to hole capture by Pr^3+^. With LuPO_4_:Ce, substantial differences in the low energy (4–25 eV) VUV excitation spectrum and PL and RL decay kinetics were observed after the NCs were annealed for 2 h at 1200°C (*vs*. at 800°C, 300°C, or unannealed), corresponding to an increase in the crystallite size from 3 nm to 35 nm. The increased size resulted in well-defined PL emission components, dramatically enhanced band-to-band excitations above ~8.7 eV, and the elimination of the slow RL decay component ascribed to surface defects. Importantly, the RL intensity for 35 nm NCs was found to be ~100× stronger than for smaller (<12 nm) NCs, whereas the PL intensity of both types was comparable.

The synthesis and characterization of a number of Pr^3+^ and Ce^3+^-activated garnet, silicate and oxide nanoscintillators have recently been reported, with an emphasis on their use for combined XRT/PDT, in particular their emission in the 300–400 nm range (Jung et al., 2014). The RL properties of powdered nanocrystalline samples prepared through combustion synthesis and annealing at 1200°C were compared with single/microcrystalline samples of similar compositions. The general composition (Y_1−*x*_Pr_*x*_)_3_Al_5_O_12_ [or yttrium aluminum garnet (YAG):Pr, with an average diameter of 80 nm] was found to have the highest scintillation yield of the nanoscintillators tested under 50 keV excitation, though with a different activator concentration dependence than single crystal samples: quenching was observed for *x* > 1%, compared to *x* = 0.16–0.65% reported for single crystals. Somewhat surprisingly, only YAG:Pr NCs with *x* = 0.75, 1, and 1.5% had greater emission intensity than Bi_4_Ge_3_O_12_ (BGO) NCs, in stark contrast to single crystals, where BGO had the lowest relative intensity of the compositions investigated. Indeed, because the RL behavior of NCs is dependent on activator concentration quenching, which is in turn dependent on the NC composition, size and crystallinity, it was suggested that the properties of different preparations will likely have to be evaluated individually rather than predicted by bulk trends. The introduction of the article also provides an inclusive overview of recent progress in nanoscintillator research for biomedical applications.

Nanoscintillators that do not emit through specific activator ions are referred to as self-activated (SA), with luminescence arising from core-valence transitions, self-trapped excitons, charge-transfer emissions or other mechanisms. YAG, BaF_2_, and Y_2_O_3_ are among those that have been adapted to the nanoscale, but have not yet been investigated to a large extent as SA nanoscintillators. These compositions are also routinely doped with other activators, resulting in various effects on their intrinsic luminescence.

### Biocompatibility of lanthanide-based materials

Preparation of LnNP bioconjugates (covalent attachment of organic molecules of interest to the NP surface ligands) appears infrequently in the literature. The principles of bioconjugation are similar to those for QDs, Au, or other NPs, with some distinct stability and solubility concerns (Cao et al., 2012; Jiang et al., 2012). Ligand-exchanged and silicated LnNPs typically present primary amine functionalities which provide some additional versatility over carboxyl groups. Amines provide a number of conjugation routes, including routine reactions with amine, isothiocyanate, carboxyl, hydroxyl, and thiol functional groups on a molecule of interest. One study reported conjugates of phosphorylethanolamine (PEA)-stabilized Eu^3+^ and Ce^3+^/Tb^3+^-doped LaF_3_ by reacting the free amine of the ligand with activated biotin-PEG or mPEG NHS esters, demonstrating a successful strategy for attachment of molecules through amide bond formation. The use of these conjugates was restricted to borate buffer. Biotin conjugates have also been prepared with CeF_3_:Tb NPs silanized using TEOS/aminopropyltriethoxysilane (APTES) (Kong et al., 2007, 2008a) and PEA-stabilized Ln^3+^-doped zirconia (Liu et al., 2012).

### Scintillating nanoparticle interactions with dyes and photosensitizers (PSs)

When nanoparticles are conjugated to PS molecules and irradiated with ionizing radiation, singlet oxygen yield will depend upon scintillation yield and energy transfer efficiency. Neither of these parameters has been widely reported in the literature. However, a good number of studies have investigated lanthanide-dye charge transfer using light excitation, and a few studies have looked at singlet oxygen generation.

Lanthanide energy and charge transfer (ET and CT) have been extensively studied for lanthanide chelates and organic dye pairs (Selvin, 1996, 2002), and more recently in LnNPs, though most efforts have focused on sensitization of 4*f*-4*f* luminescence by Ce^3+^, Yb^3+^ or surface-associated organic molecules. The situation can quickly become rather complex with lanthanides whose luminescence involves the 4*f^n^* configuration. In these cases, magnetic dipole transitions are allowed and may have intensity of the same order of magnitude as electric dipole transitions. Additionally, some induced dipole transitions are hypersensitive to the environment of the lanthanide ion and apparently follow the selection rules of electric quadrupole transitions, leading them to be referred to as pseudo-quadrupolar transitions.

A 2004 report investigated energy transfer between porous networks of interconnected 18 nm YAG:Ce^3+^ nanocrystals (NCs) and the amine-reactive fluorescent dye tetramethylrhodamine isothiocyanate (TRITC) (Wuister et al., 2004). Glycine was used to coat the NCs, bound to the surface through the carboxylate moieties and providing terminal amines for attachment of TRITC. ET for the conjugate was demonstrated through strong emission of TRITC relative to NCs following selective excitation of the NCs, as well as the appearance of a fast initial decay of the time-resolved PL. The ET was estimated using Förster-Dexter theory, giving a “critical distance” (equivalent to *R*_0_) of 7 nm, resulting in energy transfer rates of up to 10^8^ s^−1^ for Ce^3+^ sites within 5 nm of the NC surface, supposed to be ~90% of the total Ce^3+^ given the NC size.

Electrostatic complexes of CePO_4_:Tb nanorods and Rhodamine B (RhB), using Ce^3+^-sensitized Tb^3+^ emission to excite RhB, resulted in ET efficiency η up to 0.85 as determined by ratiometric luminescence analysis (Di et al., 2010). Evidence of ET was taken by the quenching of the NP steady-state luminescence and concomitant increase in RhB emission with increasing amounts of RhB. Time-resolved measurements of the ^5^D_4_ → ^7^F_5_ transition of Tb^3+^ also exhibited quenching but did not quantitatively agree, reporting efficiencies lower than those determined by steady-state quenching (η ~ 0.7 at the highest quenching condition).

A recent (2013) study investigated electrostatic complexes of LaPO_4_:Ce nanorods and the fluorescent dye coumarin 440 (C-440) using steady-state and time-resolved PL measurements (Kar et al., 2013). The Stern-Volmer sphere of action static quenching model was applied to the steady-state quenching, and the ET efficiency estimated by the ratio of the Ce^3+^ fluorescence lifetimes, giving η = 0.24 for an estimated 1:47 nanorod:dye ratio. ET was corroborated by an increase of the fluorescence lifetime of the dye, excited at 280 nm, when complexed with the nanorods.

X-ray-induced singlet oxygen production has been investigated with a handful of Tb^3+^-activated oxide and fluoride nanoscintillators coupled with grafted or encapsulated photosensitizers. In one study, 11-aminoundecanoic acid-coated La_0.8_Tb_0.2_F_3_ NPs were mixed with the water-soluble photosensitizer meso-tetra(4-carboxyphenyl) porphine (MTCP) (Liu et al., 2008), which resulted in an increase in the quenching rate of the anthracenedipropionic acid (ADPA) singlet oxygen probe compared to PS alone under 250 keV X-ray irradiation at 44 cGy/min. Singlet oxygen production was demonstrated using (Gd_0.5_Tb_0.5_)_2_O_3_ NPs with PS-encapsulating polysiloxane shells (Seve et al., 2012). The photosensitizer 5-(4-carboxyphenyl)-10,15,20-triphenyl-chlorin (TPC) was first conjugated to APTES before reaction of the TPC-APTES with TEOS for shell formation, resulting in varied amounts of covalently bound TPC embedded within the shell. In this case, increasing concentrations of encapsulated TPC resulted in quenching of the TPC PL (directly excited at 414 nm) as well as singlet oxygen production (directly detected through 1270 nm phosphorescence). This result was attributed to migration of excitation energy between TPC molecules terminated at static quenching sites, with a model developed to support the data.

Recently, energy transfer mechanisms and singlet oxygen production under optical and X-ray irradiation were studied using a similar system consisting of Gd-free Tb_2_O_3_ NPs with the photosensitizer 5-(4-carboxyphenyl)-10,15,20-triphenyl porphyrin (TPP) grafted to the polysiloxane shells after rather than during their formation (Bulin et al., 2013). The NPs and NP-PS were used in DEG solution. Upon excitation at 300 nm (primarily resulting in 4f^8^ → 4*f*^7^5d^1^ transitions in Tb^3+^), a concurrent decrease of the Tb^3+^ lifetimes (measured at 545 nm) and appearance of long PL lifetimes of the grafted PS (measured at 650 nm) were taken to be indicative of excited Tb^3+^-PS nonradiative energy transfer. Interestingly, the polysiloxane layer was implicated in the appearance of a broad emission component from the NPs with a peak ~425 nm that was also involved in efficient, fast energy transfer to TPP under optical excitation, but did not appear under X-ray excitation. Singlet oxygen yields under 44 kV X-ray excitation (from a tungsten anode, providing a dose rate of 5.4 mGy/s) were evaluated with the chemical probes singlet oxygen sensor green (SOSG) and 3′-p-(aminophenyl) fluorescein (APF). SOSG showed a steady increase in signal with both PS alone and NP-PS, with the NP-PS showing a relative increase for irradiation times >10 min. The APF probe corroborated the formation of singlet oxygen by the NP-PS system, supported by competitive quenching of singlet oxygen by addition of NaN_3_.

A small number of nanoscintillator-PS conjugate systems have demonstrated measurable enhancements of X-ray irradiation in cancer cell lines. In one study, commercially available Y_2_O_3_ NPs were modified with 2-chloroethylphosphonic acid (2-CEP) ligands, which were used to form thioether linkages to fragments of the HIV-1 TAT cell-penetrating/nuclear targeting peptide bound to the PS psoralen (Scaffidi et al., 2011). A small but significant downward trend in the growth of PS-3 prostate cancer cells with 2 Gy of 160 kVp or 320 kVp X-rays was seen as a function of particle dose. Another study reported activity of a terbium-doped gadolinium oxysulfide-Photofrin II mixture against glioblastoma cells irradiated with 120 kVp diagnostic X-rays. Radiation alone produced 20% cell suppression, and radiation plus the NP-PS combination over 90% suppression. Interestingly, the particles alone (without Photofrin) protected the cells against X-irradiation.

A theoretical paper investigated the conditions required for a nanoscintillator-photosensitizer conjugate system to produce therapeutically-relevant results, using physical parameters including nanoparticle uptake into cells, enhancement of radiation dose, scintillation light yields, and energy transfer efficiencies (Morgan et al., 2009). These parameters were used to estimate the overall singlet oxygen yield of a NP-PS system with X-ray irradiation. As singlet oxygen is considered to be the primary effector of PDT, its production was taken to be indicative of the potential of conjugates to damage malignant tissue through PS activation. Overall singlet oxygen production Φ_1O_2__ was determined from the product of the scintillation yield φ_*s*_, characteristic of the material and given in photons per MeV of absorbed radiation, the NP-PS energy transfer efficiency φ_*ET*_, and the PS singlet oxygen yield φ_*p*_. For an extremely generous value of φ_*s*_ > 10^5^ photons/MeV (derived from the energy output of bulk crystals of hygroscopic LuI_3_:Ce^3+^) and somewhat generous values of φ_*ET*_ = 0.75 and φ_*p*_ = 0.89, and using the relative X-ray absorption of the NPs, it was determined that to deliver the “Niedre killing dose” of singlet oxygen (reduction of a cell population to 1/*e* fraction, based on *in vitro* measurements of OCI-AML5 leukemia) (Niedre et al., 2002, 2003), only X-ray energies below ~200 keV (with peak efficiency ~50 keV) would be effective for reasonable total radiation doses. These results suggest that it would be difficult to produce a dramatic outcome with PDT effects alone.

It has been established that the efficacy of PDT *in vivo* depends on three primary mechanisms: direct tumor-cell killing; damage to tumor vasculature; and provocation of an immune response (in contrast to the immunosuppressive effects of radiotherapy and chemotherapy (Dolmans et al., 2003). If these observations hold true for nanoscintillator-photosensitizer systems, it is conceivable that the optimal targeting and cell-level distributions of such systems may be different from those that rely solely on radiation dose enhancement by nanoparticles (which are most effective in close proximity to cell nuclei). It would also be reasonable to expect that preserving the amphiphilicity of bioconjugated photosensitizers might be beneficial, as the tendency to associate with lipid membranes is known to be a key factor in the activity of free PS molecules (Kessel et al., 1987; Jori and Reddi, 1993). Whether active targeting to tumors improves nanoparticle accumulation in human cancers and/or treatment outcomes remains debatable. There are certainly circumstances in which passive accumulation is insufficient due to the physical properties of the tumor, but the ideal target for human tumors has not been well established (Kobayashi et al., 2013; Moghimi and Farhangrazi, 2014; Nichols and Bae, 2014).

## Other alternatives: chemotherapy-nanoparticle constructs

A large number of nanoparticle conjugates to chemotherapeutic agents have been reported, but few of these have been used for radiosensitization. This is somewhat surprising, since traditional chemotherapeutic agents often act as radiosensitizers, and probably just reflects the emerging state of the field. A few reports have targeted metal nanoparticles to cells or tumors using molecules that play an active role in destroying the target cells. In one study, radioresistant melanoma cells were exposed to Au nanorods conjugated to the RGD peptide (Xu et al., 2012). Exposure to MV X-rays decreased integrin expression and rendered the cells susceptible to radiation-induced apoptosis.

Another study showed that nanoparticle preparations of epithelial growth factor receptor (EGFR) antisense oligonucleotides radiosensitized SCCVII murine squamous carcinoma cells (Xu et al., 2012). However, the nanoparticles themselves were a delivery vehicle only, so no synergy was being sought between the particles and their cargo.

In another approach, doxorubicin conjugated to DNA-coated large Au nanoparticles was loaded into MCF-7 breast cancer cells (Starkewolf et al., 2013). Irradiation with X-rays improved cell inhibition by 33% at 10 Gy relative to Dox alone or Au nanoparticles alone. The authors attributed this observation to release of Dox by the radiation.

## Summary and conclusion

Dense inorganic nanoparticles show considerable promise for dose enhancement of radiation therapy and enabling synergistic co-treatments. Gold nanoparticles are the most studied, though are not yet in the clinic for radiation therapy. Research efforts are underway to increase the efficiency of nanoparticle-based treatments, including physical and chemical optimization of nanoparticles, improved targeting such that total doses can be reduced, and combining ionizing radiation with other therapeutic modalities. Pre-sensitization of tumors with localized heating resulting from illumination of Au nanostructures with infrared light (photothermal therapy) has shown encouraging results. A number of less expensive alternatives to Au have been produced, but have not been subject to the same level of research activity. Oxides and selenides of Pt and Bi have been shown to provide radiation dose enhancement, while those of Gd and Fe also enable magnetism-based imaging, guidance and hyperthermia.

Nanoscintillators consist of a broad class of nanostructures that emit light ranging from the ultraviolet to the infrared upon excitation by ionizing radiation, with spectra that depend primarily on composition. Energy transfer from excited state nanoscintillators to surface-attached photosensitizer molecules allows such a system to improve upon the issue of tissue transmittance encountered with typical PDT, combined with the dose enhancement provided by the dense nanoparticles. If the emitted light is of an appropriate wavelength to be absorbed by photosensitizer molecules, nanoscintillator-photosensitizer bioconjugates have the potential to improve upon the issue of tissue transmittance with typical PDT. Such systems have only recently been reported, but represent another distinct class for combined therapy that requires only ionizing radiation. As these systems have thus far only been studied *in vitro*, and cover many possible material compositions and drug varieties, it is difficult to reach definitive conclusions about their advantages and disadvantages compared to Au. While the raw materials are less expensive than Au in general, the particles tend to be less than half as dense as Au, and provide lower enhancement factors. While the surface chemistry of Au is well established and reliable, oxide and fluoride nanoscintillators have known colloidal stability issues. Certainly, if XRT and PDT effects are determined to be synergistic, such systems may soon become a viable option for nanotherapeutics.

Despite the substantial progress in nanoparticle-assisted therapies in recent years, nanoscale radiosensitization effects have not yet been studied in great detail. Further understanding of the essential principles and interactions will help establish the legitimacy of new undertakings in the burgeoning field of nanomedicine, where clinical applications are just beginning to emerge.

While a good deal of preclinical data on GNRT is available, there are not yet clinical trials in the U.S. Two types of Au nanoparticles have been FDA approved for cancer trials: Au-tumor necrosis factor conjugates (clinicaltrials.gov, NCT00356980) and Au nanoshells for photothermal therapy (AuroLase, currently recruiting, NCT01679470 and NCT00848042 for lung cancer and head and neck cancer, respectively). Hafnium oxide particles are in clinical trials as radiation enhancers (NCT01433068, currently recruiting; drug name NBTXR3).

### Conflict of interest statement

The authors declare that the research was conducted in the absence of any commercial or financial relationships that could be construed as a potential conflict of interest.
